# T2-prepared steady-state free-precession for detection of hemodynamic significance of coronary artery stenosis - a comparison to fractional flow reserve

**DOI:** 10.1186/1532-429X-15-S1-E66

**Published:** 2013-01-30

**Authors:** Thomas Walcher, Robert Manzke, Vinzenz Hombach, Wolfgang Rottbauer, Jochen Wöhrle, Peter Bernhardt

**Affiliations:** 1 Department of Internal Medicine II, Cardiology, Ulm, Germany

## Background

BOLD CMR has been shown to be able to detect myocardial perfusion differences. However, validation of BOLD CMR against FFR is lacking. Aim of our study was to analyze the potential diagnostic accuracy of blood oxygen level-dependent (BOLD) cardiac magnetic resonance imaging (CMR) in comparison to invasively measured fractional flow reserve (FFR) which served as gold standard for a hemodynamic significant coronary lesion.

## Methods

BOLD image was performed at rest and during adenosine infusion in a 1.5T CMR scanner. Thirty-six patients were analyzed for relative BOLD signal intensity increase according to the 16-segment model. Invasive FFR measurements were performed in the major three coronary arteries during adenosine infusion in all patients. A FFR ≤0.8 was regarded to indicate a significant coronary lesion.

## Results

Relative BOLD signal intensity increase was significantly lower in myocardial segments supplied by coronary arteries with a FFR ≤0.8 compared to segments with a FFR >0.8 (1.1 ± 0.2 vs. 1.5 ± 0.2, p<0.0001). Sensitivity and specificity yielded 88.2% and 89.5%, respectively.

## Conclusions

CMR BOLD imaging reliably detects hemodynamic significant CAD and is thus an alternative to contrast-enhanced perfusion studies.

## Funding

This study was partly supported by a grant of the University of Ulm (L.SBN.0050).

**Figure 1:**
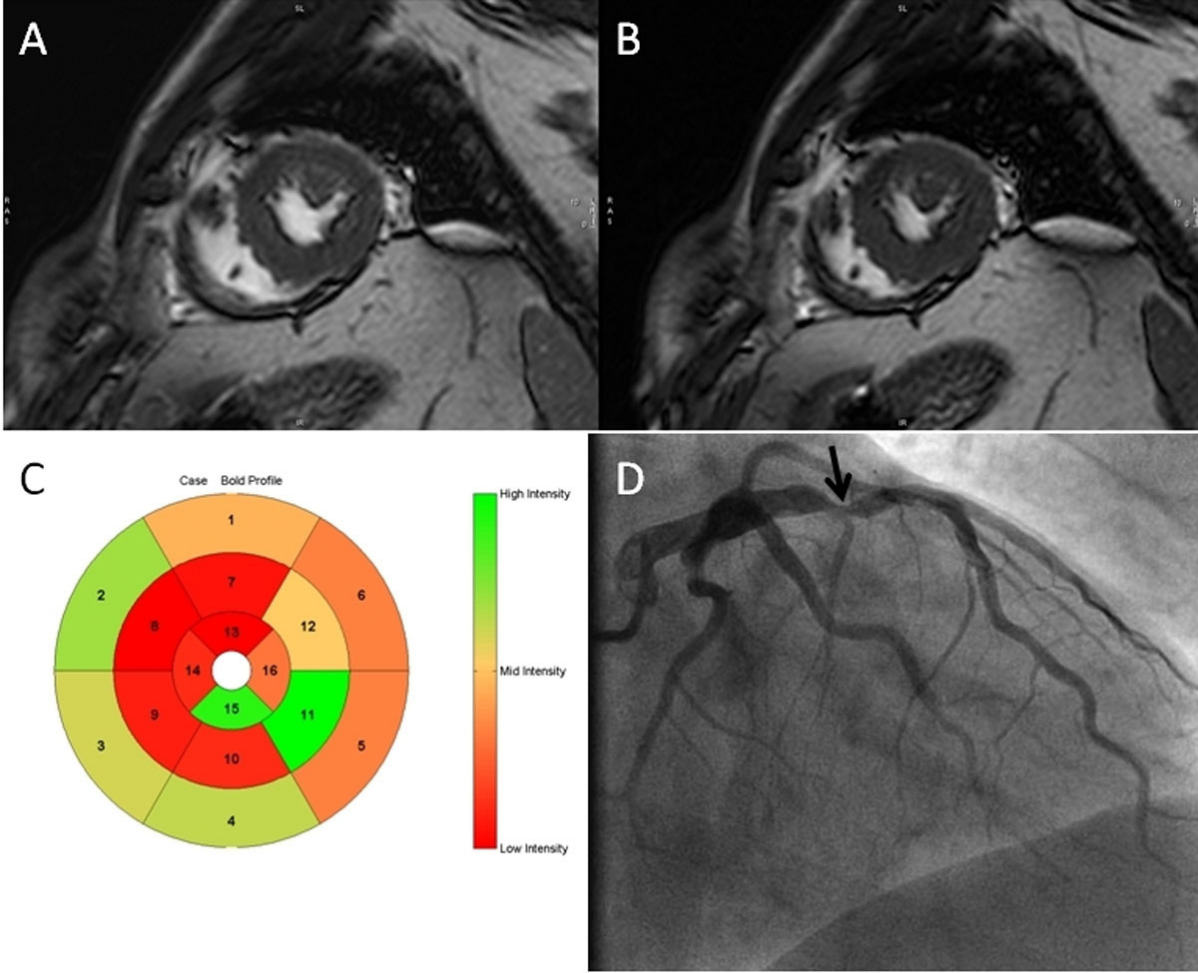
BOLD image examples in a midventricular short axis at rest (A) and during adenosine infusion (B) and color-encoded 16-segments BOLD signal intensity increase (C) in a patient with LAD stenosis (arrow) as seen on coronary angiogram (D).

**Figure 2:**
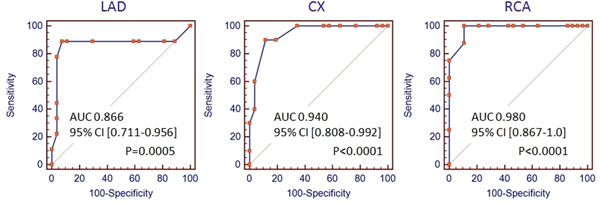
ROC curves on a per-vessel basis showing the diagnostic accuracy of BOLD CMR in LAD, CX and RCA. Area under the curve (AUC) including 95% confidence interval (CI) and p-values are provided.

